# Advancements in biomarkers for cardiovascular disease: diagnosis, prognosis, and therapy

**DOI:** 10.12703/r/10-34

**Published:** 2021-03-31

**Authors:** Nicholas Wettersten, Yu Horiuchi, Alan Maisel

**Affiliations:** 1 Division of Cardiology, Veterans Affairs Medical Center, San Diego, CA, USA; 2 Division of Cardiovascular Medicine, University of California San Diego, La Jolla, CA, USA; 3 Division of Cardiology, Mitsui Memorial Hospital, Tokyo, Japan

**Keywords:** Biomarkers, acute coronary syndrome, heart failure, prevention

## Abstract

Biomarkers are essential tools in the practice of cardiology. They assist with diagnosis, prognosis, and guiding therapy in many different cardiovascular diseases. Numerous biomarkers have become strongly associated with different cardiovascular conditions, such as troponin with acute coronary syndrome and natriuretic peptides with heart failure. Even though these biomarkers have been in practice for almost two decades, their uses continue to expand beyond their original roles. Additionally, many new biomarkers have been discovered with increasing utility in cardiovascular disease, including soluble suppression of tumorigenicity 2, galectin 3, and biomarkers of fibrosis, metabolism, and inflammation. How these old and new biomarkers are being expanded into clinical practice is constantly in evolution. This review will highlight some of the recent major advancements in the rapidly evolving field of biomarkers.

## Introduction

Biomarkers have become a backbone of risk prediction, diagnosis, and therapeutic monitoring throughout cardiology. Ever since the publication of the Breathing Not Properly trial that established B-type natriuretic peptide (BNP) as a crucial biomarker in the management of heart failure (HF), a variety of biomarkers have found increasing use in cardiology^1^. Recently, there has been a surge of studies reporting potential uses of biomarkers, both new and old. Thus, it is important for clinicians to remain up to date on the evolving uses of different biomarkers. Although many advances with biomarkers have been made throughout cardiology, such as in valvular heart disease and atrial fibrillation, this review will focus on recent advances in HF, acute coronary syndrome (ACS), and prevention. A common theme in these conditions is the substantial prognostic utility of both natriuretic peptides (NPs), BNP and its N-terminal fragment (NT-proBNP), and high-sensitivity cardiac troponin (hs-cTn). Additionally, the biomarkers soluble suppression of tumorigenicity 2 (sST2) and galectin 3 (Gal-3) are emerging with different roles in many cardiovascular conditions. Lastly, there have been many novel biomarkers for diagnosis and prognosis or to supplement current biomarkers in these areas of cardiology.

## Acute coronary syndrome

One of the most impactful biomarker advancements in the past decade has been the development of hs-cTn. Troponin is a complex with three subunits in myocytes and is involved in contraction and relaxation. Two isoforms (T and I) in cardiomyocytes are cardiac-specific and this has been exploited clinically to help identify cardiomyocyte injury with greater specificity than prior biomarkers like CK-MB. Further refinement in clinical assays has improved the sensitivity of troponin detection, and the newest generation of hs-cTn assays are able to detect troponin in at least 50% of healthy individuals; ideally, the goal is to detect troponin in more than 95% of healthy individuals^2^. The development of hs-cTn brought a new era to the diagnosis and risk stratification of patients with ACS, coronary artery disease (CAD), or other conditions. In a patient presenting with chest pain or symptoms concerning for cardiac ischemia and possible non-ST elevation (NSTE)-ACS, troponin is critical for determining the presence of myocardial infarction (MI). Although hs-cTn was rapidly adopted, questions were raised about its optimal use in NSTE-ACS. Fortunately, recent studies have reaffirmed prior findings and assured the safety of its current implementation in clinical practice. Additionally, other biomarkers have been explored in ACS that can improve the risk stratification of patients with ACS for future events.

**Figure 1.**
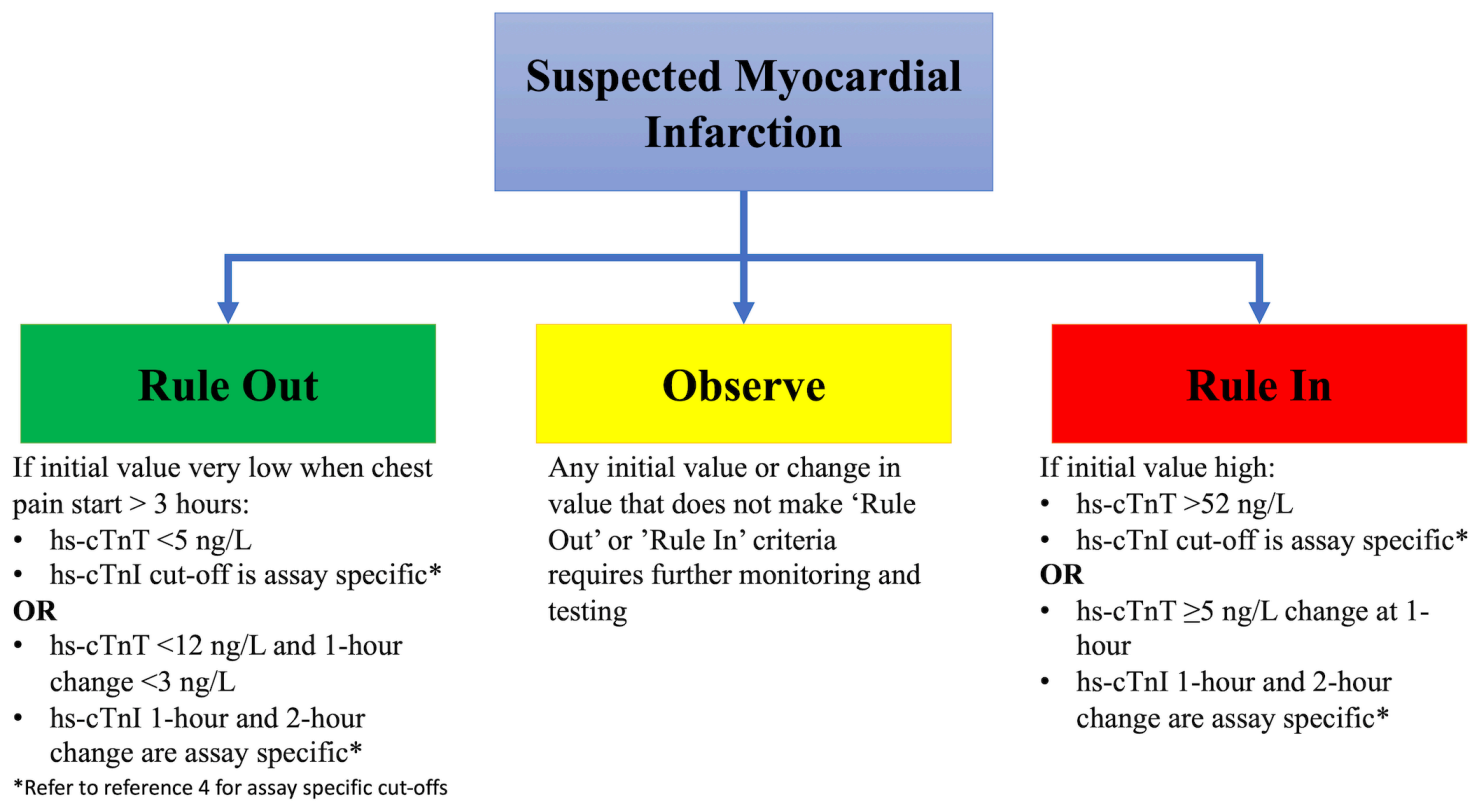
The rapid rule-out algorithm. Assessment of high-sensitivity cardiac troponin (hs-cTn) in patients presenting with suspected myocardial infarction can lead to three different pathways with either 0/1-hour algorithm or 0/2-hour algorithm. If a patient presents more than 3 hours after chest pain and has a very low hs-cTn, they can be ruled out. If the patient presents within 3 hours or does not have a very low hs-cTn, a rapid reassessment at 1 or 2 hours with minimal change in hs-cTn rules out myocardial infarction. Very high initial values or changes in values rule in a patient for myocardial infarction. For patients not meeting either criterion, further observation with serial testing of hs-cTn should be performed. Cut-offs for change at 1 or 2 hours are assay-specific. Pathway adapted from the European Society of Cardiology Guidelines.

In 2015, the European Society of Cardiology (ESC) adopted a rapid rule-out algorithm (the 0/1h-algorithm) using either hs-cTnI or hs-cTnT assessed at presentation and 1 hour later for patients presenting with suspected NSTE-ACS^3^. In 2020, these guidelines were updated to include important advances for using hs-cTn in the field of NSTE-ACS^4^. These include the incorporation of 0/2h-algorithms and recognition of vendor-specific assay cut-offs for the different hs-cTnI assay used for ruling in and ruling out acute MI (Figure 1)^4^. Initially, there was concern that the rapid algorithm lacked sensitivity and safety, but recent studies have validated the safety and utility of this algorithm as reaffirmed in the 2020 ESC Guidelines. In two prospective studies of over 6600 patients presenting with NSTE-ACS, Twerenbold *et al*. demonstrated the safety and capability of the 0/1h-algorithm^5,6^. The authors found that the 0/1h-algorithm had a negative predictive value (NPV) of more than 99.5% and patients who were ruled out for MI had very low rates of death and major adverse cardiovascular events (death and MI) at 30 days (0.1% and 0.2%, respectively)^5,6^. It is worth noting that the positive predictive value of the 0/1-h algorithm was only fair, but the power of the algorithm lies in its ability to rapidly and safely rule out an MI usually within 3 hours of presentation.

Although these studies validated the use of the 0/1h-algorithm, other important questions about the clinical use of hs-cTn in NSTE-ACS remained. The 0/1h-algorithm allows MI to be ruled out if the first hs-cTn value is below the level of detection in a patient presenting three or more hours from symptom onset. A meta-analysis demonstrated that an hs-cTnI of less than 5 ng/L using the Abbott ARCHITECT*STAT* assay had an NPV of 99.5% for excluding an MI and there were no cardiac deaths at 30 days in this population, further validating the rapid rule out of the 0/1-h algorithm^7^. Similarly, a multi-center cohort study in the US examined outcomes of patients on the basis of their initial hs-cTnI measurement using the Siemens Atellica IM and ADVIA Centaur assays and found that a level of less than 5 ng/L had a high NPV for NSTE-ACS and low risk for death^8^. Importantly, the authors also found that a cut-off of hs-cTnI of at least 120 ng/L identified patients who are at high risk for adverse cardiac events and who should strongly be considered for treatment of ACS on the basis of this single troponin measurement^8^. Recognizing the variability in assays by vendor and cut-offs proposed in prior algorithms, Neumann *et al*. went beyond prior studies and evaluated patient outcomes based on different cut-offs and time frames for repeat hs-cTn measurement^9^. In doing this, the authors developed a nomogram for risk and the NPV based on initial measurements and follow-up values^9^. This chart can be readily applied in clinical practice, helping a provider determine a patient’s risk based on their institutions cut-offs and timing of hs-cTn assessment.

A recognized analytical confounder of hs-cTn in ACS is renal impairment. Patients with renal dysfunction frequently have elevated cTn levels even if they are not presenting with ACS. This made the use of hs-cTn assays in patients with renal dysfunction a concern. However, many studies have confirmed that a low hs-cTn and the 0/1h-algorithm are still valid in patients with renal dysfunction and can safely rule out an MI^10,11^. An important caveat, though, is that far fewer patients with renal dysfunction will meet rule-out criteria since they are more frequently found to have an elevated troponin^10,11^. Additionally, the specificity of hs-cTn is lower in this population, as might be expected. Even though MI may not be subsequently diagnosed, clinicians should remember that patients with renal dysfunction have an increased risk for cardiovascular disease (CVD) and these studies found that patients with an elevated hs-cTn had a two-fold greater risk of major adverse cardiovascular events, highlighting the role of hs-cTn in risk stratification^10^.

Although studies have confirmed the utility the use of hs-cTn in renal dysfunction, there is still debate about how to account for other confounders of hs-cTn interpretation, including age and sex. Troponin values are known to increase as an individual grows older^4,12^. At least one study has shown that age-specific cut-offs can influence the diagnostic accuracy of the hs-cTnT assay, and age-specific cut-offs should be considered^13^. However, further studies are needed to validate these findings with the hs-cTnT assay and with the different hs-cTnI assays. In regard to sex, troponin values are typically lower in women than men and some hs-cTn manufacturers have recommended sex-specific cut-offs^14^. However, many studies have not shown a substantial difference in diagnostic accuracy of the hs-cTnT assay using sex-specific cut-offs^12,13,15,16^. Results with hs-cTnI assays have varied; some studies show differences in diagnostic accuracy when using sex-based cut-offs, and others show no difference^12,15,17^. Although the ESC Guidelines have not adopted age- or sex-specific cut-offs, this is still an area of active research; with further study, age- and sex-specific cut-offs may be incorporated into algorithms^4,14^. The key is for the clinician to recognize the influence of confounders such as age, sex, and renal dysfunction on diagnostic accuracy of hs-cTn. In total, though, these studies and others have solidified the safety and utility of hs-cTn to rule out MI. In the appropriate clinical setting, they provide reassurance that a patient can be discharged for further outpatient evaluation with a low likelihood of adverse outcomes.

As valuable as hs-cTn has become in ACS, it alone cannot fully risk-stratify a patient, and other biomarkers are showing promise in this area. The NPs have found an increasingly important prognostic role in ACS. NPs are released from the heart in the setting of increased wall stress and myocardial stretch most frequently associated with pressure or volume overload. NPs are frequently elevated with myocardial ischemia from increased myocardial stress and neurohormonal activation. Two recent studies have shown that patients with persistently elevated NT-proBNP following an ACS event have a substantially increased risk of cardiac death, MI, and urgent revascularization^18,19^. Notably in one of the studies, patients with elevated NT-proBNP had more cardiovascular risk factors and more frequently had left ventricular systolic dysfunction^18^. Thus, this persistent elevation in NT-proBNP may be a reflection of adverse cardiac remodeling following ischemia from increased wall tension, persistent neurohormonal elevation, or potentially even other conditions known to cause an elevation in NPs like atrial fibrillation. Thus, assessment of NT-proBNP at time of ACS and in the post-ACS period may help identify high-risk patients who might benefit from more-aggressive medical therapy and surveillance. This potentially includes aggressive up-titration of medical therapies that inhibit the sympathetic nervous system (SNS) and renin–angiotensin–aldosterone system (RAAS), control of comorbidities such as diabetes mellitus and hypertension, selection of more-aggressive lipid-lowering therapies, use of more-potent antithrombotic agents, early repeat echocardiography to assess for adverse cardiac remodeling, prolonged electrocardiographic monitoring for arrhythmias, and potentially referral for advanced therapies such as cardiac transplantation. Beyond NPs, other novel biomarkers are also showing promise for risk stratification after an ACS event.

Gal-3 is a beta-galactoside–binding lectin that is secreted by activated macrophages and reflects myocardial fibrosis and remodeling in injured myocardium. Thus, Gal-3 may add further pathophysiologic insight by reflecting cellular recruitment for repair from injury and fibrosis beyond hs-cTn which reflects cardiomyocyte injury and NPs which reflect cardiomyocyte stretch and stress. When Gal-3 was measured at the time of MI, elevated levels were associated with an increased risk of subsequent HF (hazard ratio [HR] 1.4, 95% confidence interval [CI] 1.0–2.0 for tertile 2 compared with tertile 1 and HR 2.3, 95% CI 1.6–3.2 for tertile 3 compared with tertile 1) and death (2.4-fold increased risk for tertile 3 compared with tertile 1)^20^. These risk estimates were in addition to the highest value of cTnT entered into a multivariable model demonstrating that Gal-3 was prognostic beyond cTn. Whether serial assessment enhances utility still needs to be determined. More recently, serial assessment of growth differentiation factor 15 (GDF-15), a stress-induced cytokine, in patients who present with ACS was shown to be associated with an increased risk of cardiac death, MI, and urgent revascularization in patients with persistently elevated levels^21^. In total, serial assessment of biomarkers appears to provide a powerful tool for risk stratification and potentially directing intensity of medical therapy in patients with ACS.

The field of ACS has seen many recent biomarker advancements (Table 1). The utility and safety of rapid diagnostic algorithms with hs-cTn have been robustly validated. These studies showed that hs-cTn can identify very low-risk patients for adverse cardiovascular outcomes with the improved sensitivity of these assays. However, this has come with a cost of reduced specificity as many conditions—such as HF, myocarditis, or a pulmonary embolism—may cause an elevated cardiac troponin. Identifying high-risk patients with ACS can be slightly more challenging with hs-cTn given its sensitivity, and although a more rapid increase in hs-cTn may indicate a more high-risk patient with ACS, the rise in cardiac troponin is dependent on vessel patency^22^. Other biomarkers are showing promise to improve risk stratification. NT-proBNP, Gal-3, and GDF-15 show substantial promise to further risk-stratify patients who may benefit from more-aggressive medical therapy, such as more-potent anti-platelet agents, lipid-lowering therapies, RAAS and SNS blockade, and potentially inflammation modification and surveillance. In the future, a clinician might measure Gal-3 at the time of an MI to risk-stratify patients beyond hs-cTn for risk of death and development of HF. After the ACS event, NT-proBNP and GDF-15 could be measured to further identify heightened risk for death and recurrent ACS. Whether a combination of these biomarkers could further improve risk stratification is yet to be studied, and further studies are needed to determine whether a panel of biomarkers may provide the best risk stratification of patients with ACS and which therapies to apply in high-risk patients to reduce risk of death and recurrent ACS.

**Table 1.** Biomarkers for acute coronary syndrome.

Biomarker	Use	Key points	Areas of uncertainty
hs-cTn	Diagnosis Prognosis	hs-cTnI <5 ng/L has high NPV 0/1h-algorithm and 0/2h-algorithm have high NPV Valid in renal dysfunction Age and sex influence hs-cTn levels, but age- or sex-specific cut-offs have not yet been included into Guideline-based algorithms	Identifying high-risk patients (possibly ≥120 ng/L?) Improving positive predictive value
NPs	Prognosis	Serial assessment can identify high-risk individuals	Cut-offs for high risk Frequency of monitoring What interventions to reduce risk
Galectin 3	Prognosis	Identifies increased risk of death and heart failure	Should serial assessment be performed What interventions to reduce risk
GDF-15	Prognosis	Serial assessment might identify high risk for recurrent events	Larger studies needed What interventions to reduce risk

hs-cTn, high-sensitivity cardiac troponin; GDF-15, growth differentiation factor 15; NP, natriuretic peptide; NPV, negative predictive value.

## Heart failure

One of the first areas in which biomarkers made a substantial impact in cardiology was HF. This is where BNP and NT-proBNP first demonstrated their utility for assisting with diagnosing HF, specifically with high sensitivities to exclude a diagnosis of HF. These prior findings resulted in a class I recommendation in the American College of Cardiology/American Heart Association (ACC/AHA) Heart Failure Guidelines for measuring BNP or NT-proBNP in any patient presenting with a suspected diagnosis of HF^23^. Since the adoption of NPs into clinical practice in HF, HF has remained one of the major areas of continued growth and utilization of biomarkers. Recently, the field of chronic HF has seen some exciting advancements while acute HF has also had a few major developments.

## Chronic heart failure

Prior studies of NP-guided therapy have had variable outcomes; some showed improved outcomes and others did not. Guiding Evidence-Based Therapy Using Biomarker Intensified Treatment in Heart Failure (GUIDE-IT) attempted to resolve this question with a large multi-center randomized trial comparing NT-proBNP–guided treatment versus usual care in high-risk patients with HF^24^. Patients with a left ventricular ejection fraction of not more than 40% and a prior HF event within 12 months were enrolled with the goal of reducing NT-proBNP levels to less than 1000 pg/mL by up-titration of medical therapy in those assigned to the intervention arm. The trial was stopped prior to completion because of futility. After 894 patients were enrolled, 164 patients (37%) in the biomarker-guided group and 164 patients (37%) in the usual-care arm experienced the primary composite outcome of first HF hospitalization or cardiovascular mortality (adjusted HR 0.98, 95% CI 0.79–1.22) over a median follow-up of 15 months^24^. These findings were thought to be the end of biomarker-guided therapy. However, there are important caveats to these findings and further analyses have shown the potential usefulness of biomarkers during titration of medical therapy.

**Figure 2.**
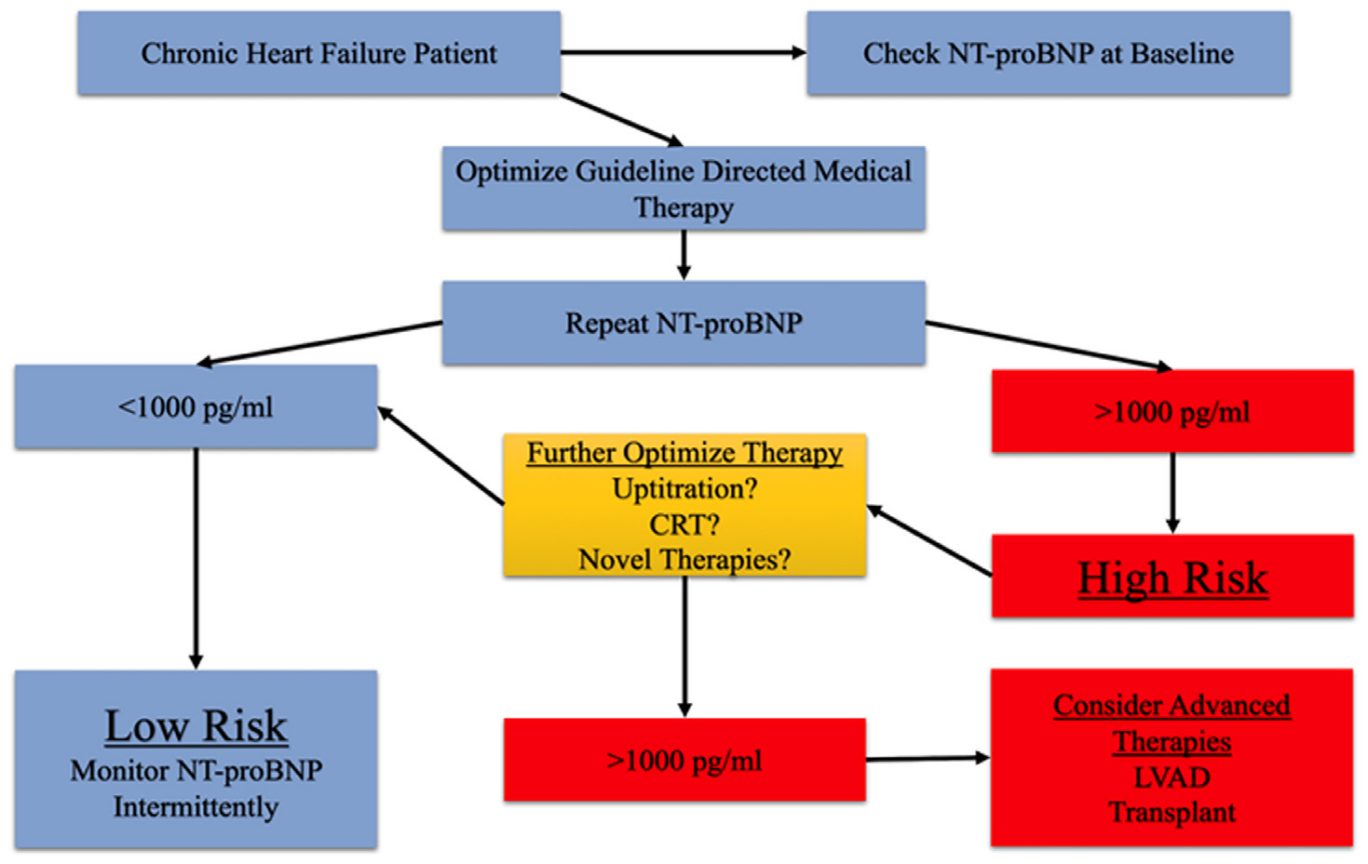
Suggested approach to natriuretic peptide–guided therapy. Patients with chronic heart failure should have an NT-proBNP assessed early in the disease course. Medical therapy should be aggressively titrated to goal dosing or highest tolerated doses. Once the patient is optimized, NT-proBNP can be reassessed. If it is less than 1000 pg/mL, the patient is low risk for adverse heart failure outcomes. If it is greater than 1000 pg/mL, attempts should be made to further optimize therapy as much as possible. If it remains greater than 1000 pg/mL, referral for evaluation of advanced heart failure, further risk stratification, and possible advanced therapies should be considered. CRT, cardiac resynchronization therapy; NT-proBNP, N-terminal pro B-type natriuretic peptide.

First, most study sites involved were HF-focused centers that routinely practiced aggressive optimization of medical therapy, which was likely applied regardless of randomization arm. Because the two strategies were similar in reaching target doses, it is not surprising that changes in NT-proBNP were similar in the two arms. Although there were more clinic visits and medication adjustments in the biomarker-guided group, up-titration of GDMT to target doses was not significantly different between the two strategies. This could be explained in part by the limited tolerability of this high-risk patient population to reach target doses. Indeed, a recent analysis of GUIDE-IT showed that patients able to achieve a reduction in NT-proBNP to less than 1000 pg/mL, regardless of randomization arm, had a substantially lower risk of cardiovascular or HF hospitalization (HR 0.26, 95% CI 0.15–0.46) and all-cause mortality (HR 0.34, 95% CI 0.15–0.77) and improved quality-of-life scores^25^. Additionally, a hypothetical experiment using the BIOlogy Study to Tailored Treatment in Chronic Heart Failure (BIOSTAT-CHF) examined outcomes in patients if they underwent three different scenarios: (1) all patients up-titrated to more than 50% of recommended doses, (2) up-titration according to a biomarker-based treatment selection model, and (3) no patient is up-titrated to more than 50% of recommended doses^26^. In these hypothetical scenarios, patients with a biomarker approach had a substantially reduced rate of death and HF hospitalization compared with other scenarios, again demonstrating the potential benefits of a biomarker-guided approach. Although these latter two analyses do suggest some potential role of biomarker-guided therapy, neither was a randomized controlled trial and it should be remembered that the only large randomized trial, GUIDE-IT, did not show benefit from a biomarker-guided strategy. With this caveat in mind, there may still be a potential role for biomarker-guided therapy. A proposed use of biomarker-guided therapy in HF is to aggressively up-titrate medical therapy in HF, checking NT-proBNP while adjusting therapy, and the most important NT-proBNP measurement is the last one measured when medical therapy is felt to be optimized. If the last NT-proBNP is more than 1000 pg/mL, then a potentially high-risk patient with HF has been identified and further means of optimization or potentially advanced therapies should be considered (Figure 2). Although we continue to propose a potential role of biomarker-guided therapy, guidelines have not yet advocated such an approach given the lack of evidence at this time.

Another consideration is the use of NPs in the setting of angiotensin receptor neprilysin inhibitor (ARNI) therapy. Neprilysin is an enzyme that breaks down many vasoactive peptides, including the various NPs such as BNP. Theoretically, inhibition of neprilysin will reduce the breakdown of BNP, causing levels to elevate in the setting of ARNI therapy while NT-proBNP levels will change independent of neprilysin inhibition as their clearance is not dependent on neprilysin, although there is debate as to whether BNP is substantially broken down by neprilysin in humans^27^. In the Prospective comparison of ARNI with ACEI to Determine Impact on Global Mortality and morbidity in Heart Failure (PARADIGM-HF) trial, ARNI therapy led to a reduction in NT-proBNP levels but did not substantially change BNP levels^28^. This finding prompted investigators to suggest that NT-proBNP should be the preferred biomarker measured in the setting of ARNI therapy. However, subsequent studies have questioned this. In a study that evaluated many assays of NT-proBNP and BNP in the setting of ANRI therapy, levels of BNP inconsistently changed depending on which assay was used, while NT-proBNP generally decreased regardless of assay used, and atrial natriuretic peptide (ANP) levels consistently increased^29^. This study highlights that BNP changes with ARNI may be more dependent on the assay used for measurement than actual neprilysin inhibition. Furthermore, an analysis of the prognostic utility of BNP with concurrent ARNI therapy from PARADIGM-HF demonstrated that BNP remained prognostic for the outcome of cardiovascular death or HF hospitalization^30^. This highlights that BNP remains a prognostic biomarker in the setting of ARNI therapy and maintains that one of BNP’s most important uses in chronic HF is for determining prognosis. Although these studies provide some evidence that the measurement of BNP with ARNI therapy is still useful, the consistent change of NT-proBNP with ARNI therapy, regardless of the assay used, makes it the preferred NP measured with ARNI therapy until further studies can better understand BNP changes with neprilysin inhibition.

Beyond NPs, hs-cTn and sST2 have affirmed their utility as important biomarkers for risk-stratifying patients with HF. Current ACC/AHA Guidelines give a class I recommendation for measuring hs-cTn in patients presenting with acute HF to evaluate for MI as a possible cause of decompensation and for risk stratification purposes; however, guidelines give only a class IIb recommendation for assessment of hs-cTn in chronic HF^31^. Mounting evidence, though, suggests a role for hs-cTn in the risk stratification of patients with chronic HF. Aimo *et al*. performed a comprehensive individual patient-level data meta-analysis of hs-cTn in patients with chronic HF^32^. It is worth noting that the majority of patients had hs-cTnT assessed given differences in assay characteristics between hs-cTnT and hs-cTnI depending on the vendor assay used (as discussed earlier). In adjusted models, hs-cTnT retained substantial risk prediction for all-cause mortality (HR 1.48, 95% CI 1.41–1.55), cardiovascular death (HR 1.40, 95% CI 1.33–1.48), and cardiovascular hospitalization (HR 1.42, 95% CI 1.36–1.49)^32^. Risk began to increase at values of more than 18 ng/L and increased further at higher levels, reinforcing the importance of checking and potentially monitoring hs-cTnT for patient risk.

ST2 is a member of the interleukin 1 (IL-1) receptor superfamily. The soluble form (sST2) is thought to be a decoy receptor for IL-33 and prevents binding of IL-33 to the ligand form of ST2. This leads to adverse cardiac remodeling, including fibrosis, hypertrophy, and apoptosis. Thus, elevated sST2 levels are associated with myocardial fibrosis and adverse cardiac remodeling. Currently, ACC/AHA HF Guidelines give measurement of sST2 a class IIb recommendation in patients with chronic HF^31^. Prior studies have shown adverse cardiovascular outcomes in chronic HF when levels are more than 35 ng/mL. Recently, Emdin *et al*. evaluated sST2 in one of the largest patient cohorts and showed that risk of all-cause death, cardiovascular death, and HF hospitalization started to rise at sST2 levels of more than 28 ng/mL^33^. Additionally, sST2 remained associated with the outcomes after adjusting for NT-proBNP and hs-cTnT levels with an HR of 1.25 to 1.3 per doubling of sST2.

Some studies also suggest that sST2 might be helpful in selecting therapies for patients with HF. In a study of 151 patients with HF with reduced ejection fraction (HFrEF), the association between beta-blocker therapy and sST2 was examined for a composite outcome of cardiovascular events^34^. Patients with high sST2 at baseline (defined as more than 35 ng/mL) and on low dose of beta-blocker during follow-up (defined as less than 50 mg equivalent dosing of metoprolol succinate) were at the greatest risk for cardiovascular events^34^. These findings suggest that beta-blocker dose should be aggressively up-titrated in patients with high sST2 levels. A study of 99 patients with HFrEF examined the association between sST2 levels and sudden cardiac death^35^. The authors found that patients with higher sST2 levels were at a greater risk for sudden cardiac death, a finding that remained significant when adjusting for NT-proBNP^35^. These findings are hypothesis-generating given the small study sizes but suggest that sST2 may be able to help risk-stratify patients for risk of sudden cardiac death and identify those most likely to benefit from implantable cardiac defibrillator therapy.

## Acute heart failure

Building on studies from chronic HF, recent studies in acute HF have applied the same biomarkers and other novel biomarkers to further improve acute HF care. Stienen *et al*. evaluated NT-proBNP–guided therapy in acute HF similar to GUIDE-IT^36^. Patients were randomly assigned to either NT-proBNP–guided therapy defined as trying to obtain a decrease in NT-proBNP of at least 30% from admission by time of discharge or usual care. In the principal analysis, no difference in all-cause mortality or HF hospitalization at 6 months was found^36^. However, most patients had already achieved at least a 30% reduction in NT-proBNP at the time of randomization, leaving only a small population to actually try to use medical therapy to guide to lower levels. A post-hoc analysis showed that patients with a 30% reduction in NT-proBNP at time of randomization had the lowest prevalence of HF readmission or death (28%) at 6 months whereas those patients who did not have a 30% reduction but subsequently achieved it with guided therapy had a higher prevalence of HF readmission or death (49%) at 6 months, but this was still lower than patients who were unable to obtain a 30% reduction in NT-proBNP who had the highest prevalence of HF readmission or death (59%) at 6 months. The authors highlighted the need to design future studies focusing on the high-risk patients who do not have a 30% reduction in NT-proBNP at the time of stability and to determine whether guided therapy can improve outcomes in this population. Thus, the utility of NP-guided therapy in acute HF is still unanswered.

The usefulness of sST2 in acute HF, as in chronic HF, was confirmed in a meta-analysis by Aimo *et al*.^37^. They found that admission and discharge sST2 values were highly prognostic for all-cause mortality and cardiovascular death, HRs ranged from 2.06 to 2.46 per doubling of sST2 for these outcomes, and discharge sST2 was predictive of HF readmission with an HR of 1.54 per doubling of sST2. van Vark *et al*. have shown that serial sST2 monitoring during and after hospitalization for acute HF can improve risk stratification^38^. They identified two patterns for sST2: a U- and a J-shaped course. J-shaped patients had high sST2 on admission for acute HF but decreased by discharge and remained low after discharge with good outcomes. U-shaped patients had a decline in sST2 that increased after discharge and was associated with an elevated risk of all-cause mortality and HF hospitalization. What drives the J- versus U-shaped trajectory in patients was not clearly defined. However, as sST2 reflects fibrosis, inflammation, and remodeling, the initial triggers leading to an HF decompensation potentially persist in those patients with a U-shaped trajectory after discharge leading to progressive HF. Thus, the trajectory of sST2 may reflect the natural trajectory of HF in each patient and it is not known whether therapeutic interventions can change this trajectory; this is an area of needed research. Similar findings might be seen with other biomarkers such as hs-cTn or NPs if serially measured, but notably the prognostic utility of sST2 measurements remained independently significant when including measurements of serial NT-proBNP in multivariate models^38^. This study ties together using sST2 in both acute and chronic HF settings and suggests that serial monitoring can enhance risk stratification. The 2017 ACC/AHA HF Guidelines have yet to make a recommendation for assessment of sST2 in the setting of acute HF.****

Since ST2 is part of the IL-1 superfamily, Pascual-Figal *et al*. recently explored whether IL-1β, an inflammatory cytokine, modulated the prognostic utility of sST2^39^. They examined sST2 and IL-1β in over 300 patients with acute HF. IL-1β levels were correlated with sST2 levels and further risk-stratified patients with an sST2 of at least 35 ng/mL by showing that only those with a concomitant elevation in IL-1β (≥49.1 pg/mL) had an increased risk of death. Further studies are needed to see whether IL-1β should always be measured with sST2 to further discriminate risk in HF.

Adrenomedullin is a vasodilatory peptide released in both acute and chronic HF and is believed to play a role in endothelial barrier function, which can be lost in a volume-overloaded state stimulating adrenomedullin production^40^. The prognostic utility of adrenomedullin in HF has been previously described but this was assessed with a stable inactive form^40^. Recently, an assay has been developed that can measure the biologically active form (bio-ADM) allowing for rapid assessment of congestion in acute HF. Two studies have evaluated bio-ADM for this role in acute HF. In the first study, bio-ADM was measured in over 2000 patients at admission for acute HF in the BIOSTAT-CHF study and separately validated in a cohort of over 1700 patients with acute HF^41^. bio-ADM was the strongest correlate with a clinical congestion score assessed at admission. Elevated bio-ADM levels were independently associated with an increased risk of all-cause mortality and HF hospitalization with an HR of 1.16 (95% CI 1.06–1.27) per log increase. In the second study, bio-ADM was measured at day 7 or discharge in over 1200 patients from PROTECT (Placebo-Controlled Randomized Study of the Selective A1 Adenosine Receptor Antagonist Rolofylline for Patients Hospitalized With Acute Decompensated Heart Failure and Volume Overload to Assess Treatment Effect on Congestion and Renal Function)^42^. bio-ADM was the strongest predictor of residual congestion at discharge, and when patients were discharged with elevated bio-ADM levels and high loop diuretic doses, they had a substantially increased risk of HF readmission with an HR of 4.02 (95% CI 2.23–7.26). bio-ADM appears to be a promising biomarker for assessing congestion and risk in patients with acute HF, but further studies are still needed to see whether risk assessment can turn into action and improved outcomes. In total, these studies demonstrate how impactful biomarkers are in the care of patients with HF (Table 2).

**Table 2.** Biomarkers for heart failure.

Biomarker	Use	Key points	Areas of uncertainty
NPs	Diagnosis Prognosis Guiding therapy	NT-proBNP persistently >1000 pg/mL identifies potential high-risk patients Repeat monitoring could identify high risk	How to further lower NT-proBNP and risk Interventions for high-risk patients Use in acute heart failure?
hs-cTn	Prognosis	hs-cTnT >18 ng/L increased risk	What interventions to lower risk? Frequency of assessment
sST2/IL-1b	Prognosis	sST2 >28–35 ng/mL increased risk Serial monitoring to identify J- versus U-shape IL-1b may modify risk	What interventions to reduce risk? Larger studies with IL-1b
bio-ADM	Prognosis Guiding therapy	Correlates with congestion severity Identify risk for death and HF hospitalization	Serial monitoring? Can it guide therapy? Prospective studies needed

bio-ADM, bioactive adrenomedullin; HF, heart failure; hs-cTn, high-sensitivity cardiac troponin; IL, interleukin; NP, natriuretic peptide; sST2, soluble suppression of tumorogensis 2.

## Prevention

One of the most exciting potentials for biomarkers is to detect patients at high risk for CVD who may be able to receive disease-modifying therapies such as lipid-lowering therapy, aggressive blood pressure control, and use of novel agents such as sodium-glucose co-transport 2 inhibitors to reduce their risk for CVD. In this area, hs-cTn has shown incredible promise. Willeit *et al.* performed a meta-analysis of 28 studies with over 154,000 patients and showed that, compared with the lowest tertile, the highest tertile of hs-cTn had relative risks of 1.43 (95% CI 1.31–1.56) for CVD (defined as coronary heart disease or stroke), 1.67 (95% CI 1.50–1.86) for cardiovascular death, 1.59 (95% CI 1.38–1.83) for coronary heart disease, and 1.35 (95% CI 1.23–1.48) for stroke^43^. Similarly, in the Atherosclerosis Risk in Communities (ARIC) study, Jia *et al*. showed that participants with no known CVD who had an hs-cTnI in the highest quintile had an increased risk of incident coronary heart disease (HR 2.20, 95% CI 1.64–2.95), ischemic stroke (HR 2.99, 95% CI 2.01–4.46), atherosclerotic CVD (HR 2.36, 95% CI 1.86–3.00), HF hospitalization (HR 4.20, 95% CI 3.28–5.37), and all-cause mortality (HR 1.83, 95% CI 1.56–2.14) compared with the lowest quintile^44^. Similar findings were recently reported with hs-cTnT, though in a smaller cohort of patients^45^. These studies demonstrate a clear potential for using hs-cTn to identify high-risk patients who may benefit from more-aggressive risk factor modification with lipid control, blood pressure control, and potentially antiplatelet agents analogous to checking a high-sensitivity C-reactive peptide for deciding intensity of lipid-lowering therapy.

While hs-cTn has demonstrated clear utility, Gal-3 has promise as a screening biomarker. Gal-3 was measured in 2477 participants in the Framingham Heart Study Offspring cohort at two time points. Changes in Gal-3 between assessments were associated with incident HF (HR 1.39, 95% CI 1.13–1.71), CVD (HR 1.29, 95% CI 1.11–1.51), and all-cause mortality (HR 1.30, 95% CI 1.17–1.46)^46^. Thus, compared with hs-cTn studies that have looked predominately at a single time point, serial Gal-3 assessment may help identify high-risk patients for incident CVD. Of note, although this study suggests that Gal-3 may help identify high-risk patients for incident HF, the use of Gal-3 in patients with symptomatic HF is limited since many previous studies have reported a lack of prognostic utility with measuring Gal-3 and medical therapy does not appear to change Gal-3 levels. Further studies are needed to determine how best to integrate these multiple biomarkers. Indeed, some studies have shown that measuring multiple biomarkers can improve risk prediction beyond current models and thus biomarker panels may be most useful in improving risk stratification^47^.

## Conclusions

Biomarkers have become an essential part of clinical practice in cardiology. The field is moving at a rapid pace, and the goal of this review is to make clinicians aware of some of the major recent advancements. A common theme is the utility of NPs, hs-cTn, sST2, and Gal-3 for risk stratification in CVD and monitoring of disease. Although many studies support the use of NPs and hs-cTn, leading to their incorporation as class I and IIa recommendations in ACC/AHA HF Guidelines for both diagnosis and prognosis, the roles of sST2 and Gal-3 are still evolving for use in risk prediction and prognosis. Furthermore, none of these biomarkers has a defined role in guiding medical therapy, although many studies have been performed with NPs and suggest potential benefit with NP guided therapy in select populations. Other novel biomarkers such as IL-1b and bio-ADM still require further study to determine a clinical role in HF, but initial study results are promising for a potential role in HF management. In the future, a multimarker approach for diagnosis and risk stratification likely will be developed to assess the complex and diverse pathophysiology of HF.

**Figure 3.**
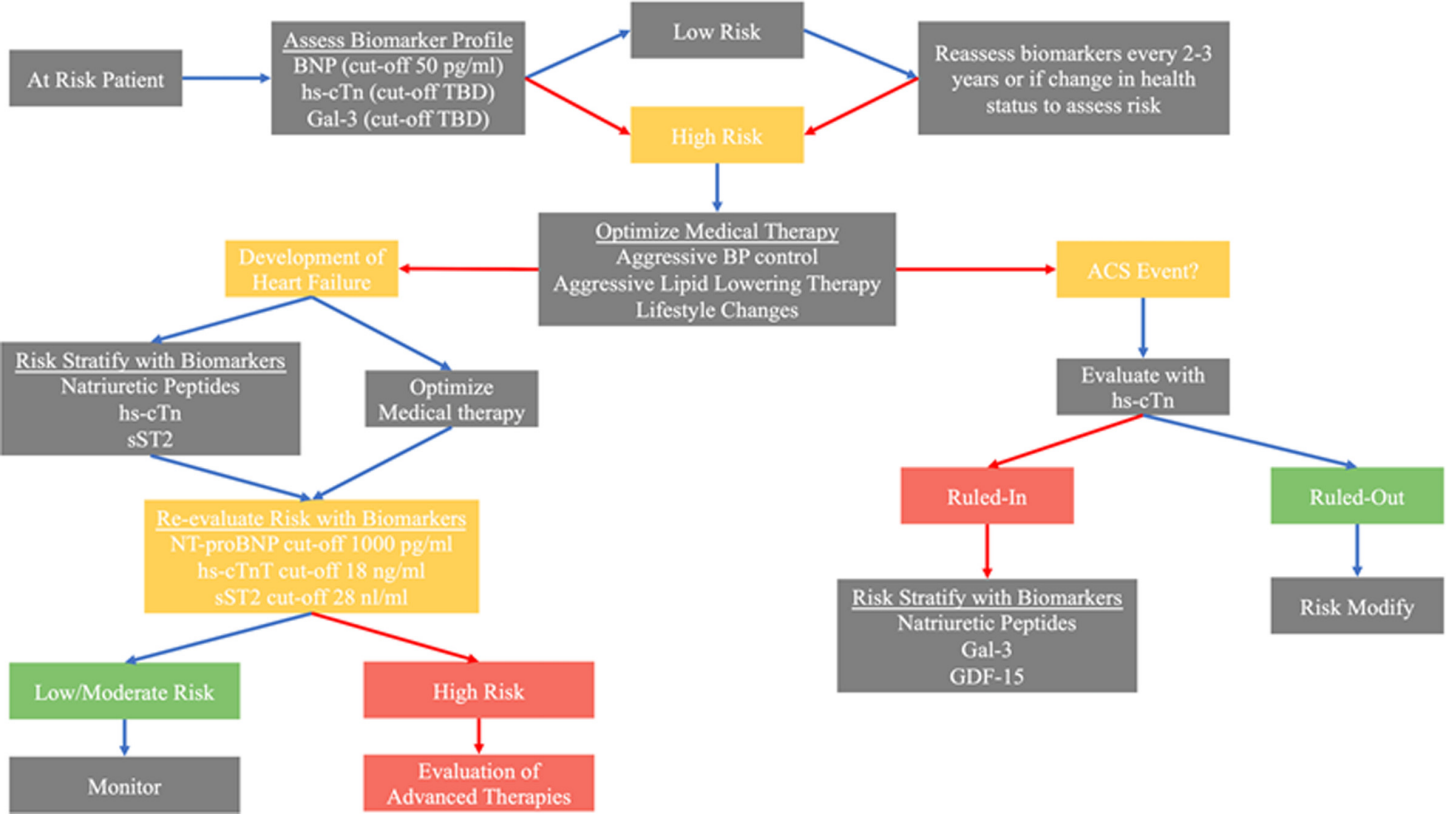
Proposed integrated biomarker pathway for prevention, diagnosis, prognosis, and therapeutic monitoring of the cardiac patient. Patients at risk for CVD should be risk stratified with BNP, hs-cTn, and Gal-3 for risk of developing CVD (that is, ACS, stroke, and heart failure). Low-risk patients should undergo intermittent reassessment of risk with biomarkers, similar to assessing hemoglobin A1c in patients at risk for diabetes mellitus. High-risk patients should be provided aggressive disease-modifying therapies. If an ACS event is suspected to occur, hs-cTn can be used to rule in or out a myocardial infarction. In those with myocardial infarction, assessment of natriuretic peptides, Gal-3, and potentially GDF-15 can identify high-risk individuals for more-aggressive therapy and surveillance. If a patient develops heart failure, patients should be risk-stratified with natriuretic peptides, hs-cTn, and sST2 before and after optimization of medical therapy. Those with biomarkers persistently above cut-offs should be considered high-risk and potentially referred to an advanced heart failure center for further evaluation and consideration for advanced therapies. BNP, B type natriuretic peptide; Gal-3, galectin 3; GDF-15, growth differentiation factor 15; hs-cTn, high-sensitivity cardiac troponin; NT-proBNP, N-terminal proBNP; sST2, soluble suppressor of tumorigenicity 2; TBD, to be determined.

The integration of these various biomarkers into clinical practice can be challenging. A proposed algorithm for using these biomarkers in the disease course of a patient is presented (Figure 3). Cut-offs for BNP, NT-proBNP, hs-cTnT, and sST2 in the proposed algorithm are based on individual studies with large patient cohorts but these cut-offs would benefit from further confirmatory studies^24,32,33,48^. Other promising novel biomarkers have been mentioned and may become more widely used clinically in the years to come but lack enough evidence for current incorporation into practice. Clinicians should maintain vigilance for future developments with these biomarkers and others.
